# Effects of Cognitive Control Exertion and Motor Coordination on Task Self-Efficacy and Muscular Endurance Performance in Children

**DOI:** 10.3389/fnhum.2018.00379

**Published:** 2018-09-24

**Authors:** Jeffrey D. Graham, Yao-Chuen Li, Steven R. Bray, John Cairney

**Affiliations:** ^1^INfant and Child Health (INCH) Lab, Department of Family Medicine, McMaster University, Hamilton, ON, Canada; ^2^Institute of Population Health Sciences, National Health Research Institutes, Miaoli, Taiwan; ^3^Department of Kinesiology, McMaster University, Hamilton, ON, Canada; ^4^Faculty of Kinesiology and Physical Education, University of Toronto, Toronto, ON, Canada

**Keywords:** self-control, self-regulation, motor control, motor behavior, mediation

## Abstract

Emerging research shows a strong connection between brain areas governing cognition and motor behavior. Indeed, research based on the Strength Model has shown that people perform worse on physical tasks following the exertion of high (compared to low) cognitive control which has been attributed to the dysregulation of neurophysiological processes within areas of the brain responsible for cognition. Yet, research investigating the negative aftereffects of high cognitive control (HCC) exertion on task performance has not considered the potential role of areas governing motor behavior. The present study investigated the effects of HCC exertion on task self-efficacy and exercise performance in children. A secondary purpose was to investigate whether motor coordination influences the change in exercise performance differently following low versus HCC exertion. Participants (*N* = 70) performed two isometric handgrip endurance trials separated by a Stroop task, which was either congruent low cognitive control (LCC) or incongruent (HCC). Motor coordination was assessed prior to the first endurance trial. Task self-efficacy for performing the second endurance trial was assessed following the Stroop task. Participants in the HCC condition reported lower task self-efficacy and showed a reduction in endurance exercise performance. Task self-efficacy mediated the cognitive control—performance relationship. Participants scoring lower on motor coordination showed the greatest declines in exercise performance following HCC, whereas motor coordination did not affect performance following LCC. The results of this study provide evidence that task self-efficacy and exercise performance are also negatively affected in children following HCC, and interestingly, these effects are exacerbated among those scoring lower in motor coordination. We recommend future research investigate motor coordination as a potential mechanism for the reductions in both cognitive and physical task performance following the prolonged exertion of HCC.

## Introduction

Cognitive control refers to “*mental processes that allow behavior to vary adaptively depending on current goals*” (Inzlicht et al., 2015, p. 126). These mental processes primarily include the self-regulation and self-control of behavior, which are governed by brain regions responsible for executive functioning (Miyake et al., 2000; Hofmann et al., 2012). Cognitive control has been implicated in various adaptive behaviors including healthy eating, weight control, and school and work performance (de Ridder et al., 2012). Emerging research also highlights the importance of cognitive control for sport and exercise performance (for reviews see Englert, 2016, 2017) as well as participation in physical activity (Buckley et al., 2014). However, exerting cognitive control is effortful and failures are abundant (Baumeister et al., 1994).

The Strength Model (Baumeister et al., 1998; Baumeister and Vohs, 2016b; Baumeister et al., 2018) posits that the resources responsible for cognitive control are finite and become fatigued with use. As such, the model has inspired hundreds of studies highlighting the negative aftereffects of high cognitive control (HCC) compared to low cognitive control (LCC) exertion on task performance across a diverse range of behaviors (for meta-analyses see Hagger et al., 2010; Cunningham and Baumeister, 2016). Studies examining the aftereffects of varying degrees of cognitive control over successive tasks (i.e., two or more tasks performed one after another) have employed the dual, or sequential, task paradigm. For example, after participants exerted cognitive control over their emotions (i.e., suppressing feelings of disgust) for an extended period of time (e.g., 5-min) they showed poorer physical task performance when compared to participants who were able to freely express their emotions (Wagstaff, 2014). Other studies have shown, following the exertion of high cognitive control (HCC), that people have lower pain tolerance, procrastinate more, and persist for less time on difficult or unsolvable tasks (Vohs et al., 2008), dieters eat more unhealthy foods (Vohs and Heatherton, 2000), and people make more impulsive choices when spending money (Vohs and Faber, 2007). However, the majority of findings are limited to young adults.

As far as we are aware, only four studies have examined the effects of HCC exertion on task performance in children. Gunzenhauser and von Suchodoletz (2014) showed that children performed worse on a cognitive inhibition task following the exertion of emotional cognitive control. Price and Yates (2010, 2015) found that children chose easier math problems (2010), were less creative (2015) and showed poorer quality in their school work (2015) following HCC exertion. Recently, Englert and Bertrams (2017) found that children’s academic performance (i.e., knowledge retrieval) was negatively affected following HCC exertion. Although findings from these studies show that children are also susceptible to the negative aftereffects of cognitive control exertion, there has been no research examining the effects of cognitive control exertion on exercise performance in children.

Cognitive control abilities assessed in childhood are predictive of several adaptive physical, social, and mental health outcomes up to 40 years later (Mischel et al., 2011; Moffitt et al., 2011). This research highlights the importance of developing cognitive control abilities in early childhood. One strategy to enhance (or develop) cognitive control in children is through regular participation in physical activity (Hillman and Biggan, 2017). However, there is a global physical inactivity epidemic occurring among children (Tremblay et al., 2014; Sallis et al., 2016) making them susceptible to various negative health outcomes as they age. Thus, research investigating the effects of cognitive control exertion on physical activity behavior among children serves as a potential new avenue for understanding inactivity patterns recently observed among young adults (Pfeffer and Strobach, 2017; Rebar et al., 2018).

Although there is an ongoing theoretical debate regarding the nature of resources or processes governing cognitive control (e.g., Beedie and Lane, 2012), with proponents advocating the role of motivation (Inzlicht et al., 2014; Inzlicht and Schmeichel, 2016), recent work highlights an influential role for task self-efficacy. Task self-efficacy refers to one’s beliefs in their abilities to perform a specific task (Bandura, 1997). Studies have found that participants consistently report lower task self-efficacy prior to completing a range of tasks (e.g., resistance exercise, isometric handgrip endurance, arithmetic) after having exerted cognitive control on an earlier, unrelated, task (e.g., Stroop task) (Chow et al., 2015; Graham and Bray, 2015; Brown and Bray, 2017b; Graham et al., 2017). Importantly, performance on the later task was impaired following cognitive control exertion and task self-efficacy statistically accounted for (i.e., mediated) the negative changes in task performance. Self-efficacy is a strong and reliable predictor of behavior including sport and exercise (Bandura, 1997; McAuley and Mihalko, 1998; Moritz et al., 2000) and has been repeatedly found to be the one of the strongest predictors of physical activity behavior in children and adolescents (Sallis et al., 2016). Thus, self-efficacy should be considered when investigating the effects of cognitive control exertion on exercise performance among children.

Despite the ongoing debate regarding the resources (or processes) governing cognitive control, there is now substantial evidence supporting the critical role of the prefrontal cortex, in addition to other regions within the frontal lobe, for enabling effective cognitive control (Wagner and Heatherton, 2016). However, reviews of neuroimaging studies also highlight the strong connection between brain areas responsible for cognition and motor behavior (Diamond, 2000; Leisman et al., 2016; Munro et al., 2017). For instance, when performing tasks requiring cognitive control, areas of the brain governing motor coordination (i.e., cerebellum and motor cortex) are also activated alongside areas governing cognitive control (i.e., prefrontal cortex). This pattern of co-activation suggests brain regions responsible for cognitive control and motor behavior may communicate to facilitate both cognitive and motor task performance. In other words, the negative aftereffects of HCC may not only be attributable to alterations in neurophysiological processes within the frontal lobe but may also be due to alterations within motor areas and, ultimately, the communication between these regions. Furthermore, Pangelinan et al. (2011) showed, in typically developing children, that performance on both cognitive and motor tasks is directly related to the brain structure (i.e., gray and white matter volume) of regions governing cognition and motor behavior. Recent research has also provided evidence that motor abilities are related to cognitive performance (i.e., academic achievement) through the mediation of executive functioning (Schmidt et al., 2017). Yet, as far as we are aware, research based on the Strength Model investigating the neurophysiological contributions leading to cognitive control failures over successive tasks has not considered areas of the brain responsible for motor behavior.

The close connection between cognition and motor behavior is also a fundamental tenant in the theory of embodied cognition and critical during the developmental period from childhood to adolescence (for reviews see Smith and Gasser, 2005; Koziol et al., 2012). Indeed, areas of the brain associated with cognition and motor behavior develop rapidly and concurrently during childhood (Diamond, 2000; Casey et al., 2005). The notion that the communication between the prefrontal cortex and other brain regions is disrupted following HCC has also been recently proposed (cf. Baumeister and Vohs, 2016a, p. 575; also see Kelley et al., 2015). Thus, based on the literature reviewed above, it seems plausible that motor coordination abilities may affect the cognitive control—performance relationship in children.

The overarching objective of this study was to investigate the effects of cognitive control exertion on task self-efficacy and exercise performance in children. Consistent with the literature reviewed above, we hypothesized that HCC exertion would negatively affect task self-efficacy and exercise performance. We also hypothesized that task self-efficacy would mediate the effect of cognitive control exertion on exercise performance. A secondary objective was to investigate whether children who score higher and lower on motor coordination would perform differently on the exercise task following low versus HCC exertion. We hypothesized children scoring lower on motor coordination (LMC) would experience greater negative effects on exercise performance following HCC exertion compared to those with high motor coordination (HMC).

## Materials and Methods

### Participants and Design

**FIGURE 1:**
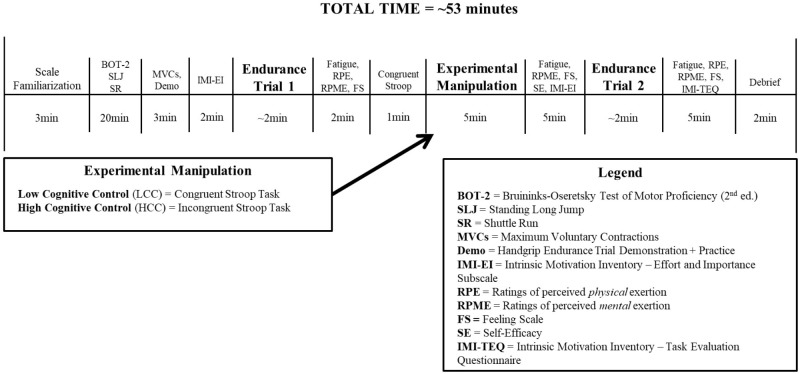
Study timeline and protocol.

Participants were 70 children (*n* = 28 females; age range: 7–14; *M*_age_ = 10.14 ± 1.90) who were enrolled in a 2-week recreational summer sport day camp at a university and were tested in a lab at the same institution. The study utilized a single-blind, randomized experimental design with one independent variable: group, consisting of two levels—LCC exertion/HCC exertion; and two dependent measures: task self-efficacy and endurance exercise performance. The primary hypotheses related to expected changes in task self-efficacy and endurance exercise performance. For an overview of the experimental protocol as well as a timeline of manipulations and measures see **Figure 1**.

The effect sizes reported below, calculated using means and SDs, are based on Cohen (1992) and the values for small, medium, and large are, respectively, 0.20, 0.50, and 0.80. A sample-size calculation (G^∗^Power version 3.1.9.2; Faul et al., 2009), based on medium-large effect sizes for task self-efficacy (*d*s ranging from 0.72 to 1.22, Graham and Bray, 2015; Graham et al., 2017), changes in endurance exercise performance (*d*s ranging from 0.80 to 1.28; Graham and Bray, 2015; Graham et al., 2017) and cognitive control task performance in children (*d*s ranging from 0.50 to 0.97, Gunzenhauser and von Suchodoletz, 2014; Price and Yates, 2015) following HCC exertion with power = 0.80 and α = 0.05, indicated a sample of *N* = 68 was sufficient for the analysis. The sample-size calculation (G^∗^Power version 3.1.9.2; Faul et al., 2009) for the proposed mediating effect of self-efficacy was based on medium-large effect sizes (Graham and Bray, 2015; Graham et al., 2017), power = 0.80, and α = 0.05, indicated a sample of *N* = 42 was sufficient for the analysis. The secondary hypothesis investigating the potential effect of motor coordination involved a 2 (LCC/HCC) × 2 (LMC/HMC) univariate analysis of variance (ANOVA). Given the lack of previous research investigating the effects of motor coordination on performance following HCC, no effect size could be determined for a sample-size calculation. Therefore, the secondary analyses were considered exploratory.

The present study was approved by the Ethics Research Committee of McMaster University in accordance with the ethical standards of the Declaration of Helsinki Rickham (1964). All participants received verbal and written information about the main characteristics of the study. Written parental consent and child assent was obtained prior to beginning the study.

### Experimental Manipulation

Participants completed two isometric endurance trials separated by manipulations of HCC and LCC. Following the first endurance handgrip trial, participants completed either a modified incongruent Stroop color word task (HCC) or a congruent Stroop color word task (LCC) for 5 min. However, prior to being randomized (stratified by sex) into their experimental condition using a random number generator^1^, participants performed the congruent version of the Stroop task for 1 min and were asked to read aloud the words they saw printed. Based on recent recommendations for cognitive control manipulations (Baumeister and Vohs, 2016a), we wanted participants to initially form the habit of reading the words presented to them so that those who were randomized to the HCC condition would then have to override the impulse of reading the word presented and instead say the name of the color they saw.

The incongruent version of the Stroop task (Stroop, 1935) is designed to be more challenging, requiring greater inhibition and processing demands than the congruent version (for a theoretical review see MacLeod, 1991), and has been supported through meta-analysis as an effective cognitive control manipulation (*d* = 0.40, Hagger et al., 2010). However, previous work has shown that younger children may show less of an interference effect than older children and adults, suggesting that they may experience less cognitive demands when performing the incongruent version of the Stroop task (see MacLeod, 1991, “*Age Differences*,” pp. 184–185). As such, pilot testing was conducted with the incongruent version of the Stroop task to investigate whether children in a sample equivalent to our sample (i.e., randomly selected from the same summer day camp) perceived the incongruent version of the Stroop task to be more challenging than the congruent version. Results of the pilot study revealed that children (*N* = 30, *M*_age_ = 9.87 ± 1.38) rated the incongruent version of the Stroop task to be more challenging, with the 76% of children rating it as “*a lot harder*” and 24% rating it as “*a little harder*.”

#### HCC Task

For the incongruent Stroop task (Stroop, 1935), participants were presented with lists of words printed on laminated sheets of 8.5 × 14-inch paper in which the print ink color and printed text were mismatched (e.g., ink color was “yellow” and the word text read “green”). Participants were required to say aloud the color of the print ink and ignore the text for each word presented. The incongruent Stroop task has been used in numerous investigations as a cognitive control manipulation (e.g., Bray et al., 2008) and has shown reliable, medium-sized, effects (Hagger et al., 2010).

#### LCC Task

For the congruent Stroop task (control), participants were presented with a list of words in which the print ink color and printed text were matched. Participants were asked to read aloud the word presented.

### Measures

#### Exercise Performance

The primary dependent measure was the change in the amount of time (between two endurance trials) participants maintained an isometric endurance handgrip contraction at 30% of their maximum voluntary contraction (MVC) using their dominant hand.

Prior to the first endurance trial, participants performed 2, 4-s 100% MVCs (separated by 2 min) using a handgrip dynamometer (model MLT003/D; ADInstruments, Colorado Springs, CO, United States) with graphic computer interface (PowerLab 4/25T; ADInstruments, Colorado Springs, CO, United States). The average force recording obtained from a 1-s window at the peak of each MVC was analyzed to determine peak force generation. The greatest peak force value was used to determine the 30% MVC target value for the endurance trials. The target force was shown as a static red horizontal line on a 17-inch computer monitor. The experimenter performed a 10-s demonstration of the endurance task, which was followed by a 10-s practice trial by the participant. This was done to ensure that the participants understood the task requirements and clarification (or another brief demonstration) was provided if necessary.

In order to perform the endurance task, participants squeezed the handgrip dynamometer and were provided with visual feedback on the computer monitor in the form of a force tracing line (i.e., a real-time graphed line which indicated how much force was being generated). Participants were instructed to sustain a handgrip squeeze for as long as possible that kept their active force tracing line at, or slightly above, the static 30% criterion line. If the force tracing fell below the 30% criterion, participants were instructed to “squeeze harder so the line stays above the marker on the screen.” The trial ended when the active force tracing line fell below the criterion line for longer than 2 s despite corrective feedback or when participants voluntarily stopped. The experimenter followed a script and no verbal encouragement or motivational feedback was provided at any time. Participants had no knowledge of the magnitude of force generation or elapsed time during the endurance trials.

#### Task Self-Efficacy

Self-efficacy for task performance on the second endurance trial was assessed using a four-item scale adhering to recommendations by Bandura (2006) for assessing self-efficacy. Each item was prefaced with the stem “*I am confident that I can hold the handgrip for…”*. The individual items represented gradations of performance that were relative to the participant’s performance on the previous trial. The scale began at (1) “*Almost as long as last time*” followed by (2) “*As long as last time*,” (3) “*A little bit longer than last time*,” and (4) “*A lot longer than last time*.” Participants rated their confidence for each item using an 11-point, 0 (*not at all confident*) to 10 (*totally confident*), scale. The task self-efficacy score was computed by averaging the ratings for each interval score to produce a scale value out of 10. Internal consistency of the scale was acceptable (Cronbach’s α = 0.75).

#### Motor Coordination

The Bruininks-Oseretsky Test of Motor Proficiency, 2nd Edition—Brief Form (BOT-2, Bruininks and Bruininks, 2005) was used to assess motor coordination. The BOT-2 consists of 12 tasks involving fine (e.g., drawing a line through a path, stringing blocks) and gross (e.g., one-handed catch, dribbling a ball) motor skills. The BOT-2 takes approximately 15–20 min to administer. Total point scores were calculated by summing the scores for each item and were then converted to sex-specific standardized scores to control for age and sex.

#### Ratings of Perceived Physical Exertion

Following each endurance handgrip trial, participants rated their perceived *physical* exertion (RPE) using Borg’s CR-10 scale (Borg, 1998) in order to determine the extent to which they exerted their physical maximum effort on each trial. Participants were instructed to rate their perception of physical exertion from 0 (*no exertion at all*) to 12 (*absolute maximum*), with 10 (*extremely strong*) representing the highest physical exertion they had ever experienced.

#### Ratings of Perceived Mental Exertion

Following each endurance trial and the Stroop task, participants rated their perceived *mental* exertion (RPME) using an adapted version of Borg’s (1998) CR-10 scale. This was done to determine the effectiveness of the cognitive control manipulation for requiring mental effort. The RPME scale was also used to determine the amount of mental effort required to perform the endurance handgrip trials. Participants were asked to indicate how much mental effort was required to perform each task and rated their effort on the scale ranging from 0 (*nothing at all*) to 12 (*absolute maximum*), with 10 (*extremely strong*) representing the highest mental exertion they had ever experienced. Numerous studies have used this version of Borg’s (1998) CR-10 scale when assessing perceived mental exertion (e.g., Bray et al., 2012).

### Potential Covariates

#### Muscular Strength and Body Composition

Standing long jump, grip strength (MVC), and the 50-foot shuttle run (taken from the BOT-2 Complete form) served as indicators of muscular strength (Bruininks and Bruininks, 2005; Castro-Piñero et al., 2010). Body composition was represented by calculating body mass index through measurements of height and weight.

#### Executive Functioning

The Behavior Rating Inventory of Executive Function, Second Edition (BRIEF2)—Parent Form (Gioia et al., 2000) was used to assess trait levels of cognitive control (e.g., inhibition, emotional control, working memory). The parent form consists of 63 items rated on a 3-point Likert-type scale ranging from 1 (*Never*) to 3 (*Often*). An example item is: “*Does not think before doing* (*is impulsive*).” BRIEF2 scores were calculated for three indexes—the Behavior Regulation Index (BRI), the Emotion Regulation Index (ERI), and the Cognitive Regulation Index (CRI)—and an overall summary score, the Global Executive Composite (GEC). Scores were converted to standardized scores (i.e., *T* scores) to control for age and sex. The measure demonstrated excellent internal consistency (α = 0.95).

#### Motivation

Motivation for performing the handgrip endurance task was assessed using two measures. Immediately prior to each endurance trial, participants completed the five-item effort and importance subscale from the Intrinsic Motivation Inventory (IMI; Ryan, 1982). The effort and importance subscale is a five-item 7-point Likert-type scale ranging from 1 (*not at all true*) to 7 (*very true*). Each item was prefaced with the following stem “*For the handgrip squeezing task I am about to do*.” An example item is: “*I am going to put a lot of effort into this*.” Internal consistency estimates for the pre-task scales were good (α’s > 0.83).

Following the completion of the second endurance trial, participants completed the IMI Task Evaluation Questionnaire (Ryan, 1982). The Task Evaluation Questionnaire consists of 22 items rated on a 7-point Likert-type scale ranging from 1 (*not at all true*) to 7 (*very true*). An example item is: “*I enjoyed doing the task very much*.” The questionnaire has four subscales: interest/enjoyment, perceived choice, perceived competence, and pressure/tension. The measure demonstrated good internal consistency (α = 0.81).

An alternative account to the Strength Model (Baumeister and Vohs, 2016b) suggests that the negative aftereffects of HCC exertion on performance are primarily attributed to changes in perceptions of fatigue, negative affect, and motivation (Inzlicht et al., 2014; Inzlicht and Schmeichel, 2016). Therefore, we wanted to ascertain that any change in endurance performance was not due to shifts in fatigue, affect, and motivation caused by the Stroop task and to control for these effects if they were evident in the analyses (i.e., potential covariates).

#### Affect

Following each endurance trial and the Stroop task, participants rated their affective valence using an adapted version of Hardy and Rejeski (1989) Feeling Scale. The Feeling Scale is a single-item measure that is scored on an 11-point bipolar scale ranging from -5 (*Very Bad*) to +5 *(Very Good*). The Feeling Scale was originally developed to assess affect during exercise, but was adapted to assess affect immediately following physical (i.e., handgrip) and cognitive (i.e., Stroop) task performance. Participants were asked to verbally report a number corresponding to their current feeling state.

#### Fatigue

Following each endurance trial and the Stroop task, participants rated their perceptions of fatigue using a Visual Analogue Scale (Wewers and Lowe, 1990). Participants were asked to rate “*how tired or energized you feel at this moment*” by drawing a vertical line through a 100-mm line with the anchors ranging from “*Extremely Tired*” on the left hand side corresponding with 0, and “*Extremely Energized*” on the right hand side corresponding with 100. Scores were calculated from 0 to 100 by measuring the distance in millimeters the participants’ vertical line was placed from the left side of the scale.

### Procedure

The study protocol and timeline is presented in **Figure 1**. Prior to taking part in the study, parents of the participants provided informed consent and completed the BRIEF2. Upon entering the lab, assent was obtained and the parameters of the study were explained. The BOT-2 was then administered, followed by two standing long jumps and then two 50-foot shuttle runs. Participants were then introduced to the handgrip task and completed two 100% MVC handgrip squeezes separated by 2 min of rest. Following the MVCs, the experimenter setup up the feedback monitor with a static horizontal red line showing the 30% MVC target value and provided a 10-s demonstration of the task. Participants then performed a 10-s practice trial to familiarize themselves with the task.

There was a 2-min rest period following the practice trial in which participants completed the pre-task effort and importance IMI subscale. Participants then completed the first endurance trial with the instructions to hold the contraction for as long as possible and *resist the temptation to quit*. After finishing the first endurance trial there was a 2-min interval period before the Stroop task and participants were asked to provide ratings on the fatigue scale, RPE scale, RPME scale, and the Feeling Scale. They were then randomized (stratified by sex), using a random number generator, to either the HCC or LCC conditions.

Participants completed the congruent version of the Stroop task for 1 min and then provided a rating on the RPME scale. Participants then completed their respective experimental manipulation task for 5 min (incongruent Stroop task or congruent Stroop task) after which they provided ratings on the fatigue scale, RPME scale, and the Feeling Scale. There was then 5 min of rest in which all of the participants completed the task self-efficacy rating scale and the pre-task effort and importance IMI scale. Participants then completed the second endurance trial with the same instructions as the first. After finishing the trial, participants provided ratings on the fatigue scale, RPE scale, RPME scale, and the Feeling Scale. They then completed the IMI Task Evaluation Questionnaire prior to being debriefed and remunerated $15 (Indigo gift card). The total time to complete the study was approximately 53 min per participant.

## Data Analysis

All statistical analyses were conducted using SPSS 25. Descriptive statistics were computed for all study variables. Separate one-way ANOVA models were computed to assess differences in means between conditions for age, potential covariates, manipulation checks (Stroop task performance and RPME scores), and ratings of perceived exertion (RPE and RPME) for exercise performance. The main hypotheses predicting between-group differences in self-efficacy and exercise performance (time to failure change) were evaluated using one-way ANOVAs.

Residualized change scores for time to failure were also analyzed. Residualized change scores have been used in addition to raw change scores to determine endurance performance effects following HCC exertion (e.g., Bray et al., 2008; Graham and Bray, 2015) and were calculated by regressing the Trial 2 contraction duration on the Trial 1 contraction duration (Cohen et al., 2003). Residualized change scores were calculated because they control for the negative correlation between baseline scores and raw change scores, as individuals who hold longer muscular contractions tend to have larger trial-to-trial changes.

**FIGURE 2:**
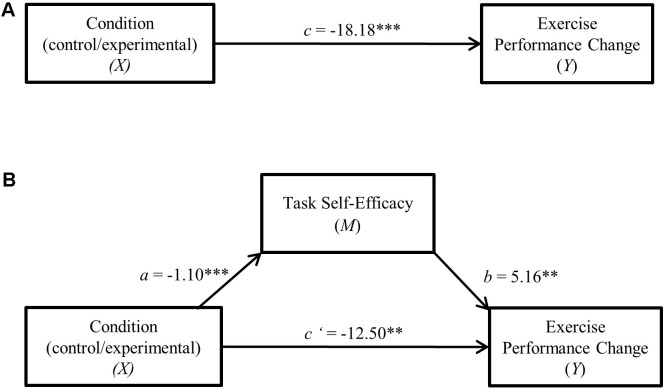
**(A)** Direct effect of cognitive control exertion on raw time to failure change. ^∗∗∗^*p* < 0.001. **(B)** Cognitive control exertion—raw time to failure change single mediation model. ^∗∗^*p* < 0.01, ^∗∗∗^*p* < 0.001.

**FIGURE 3:**
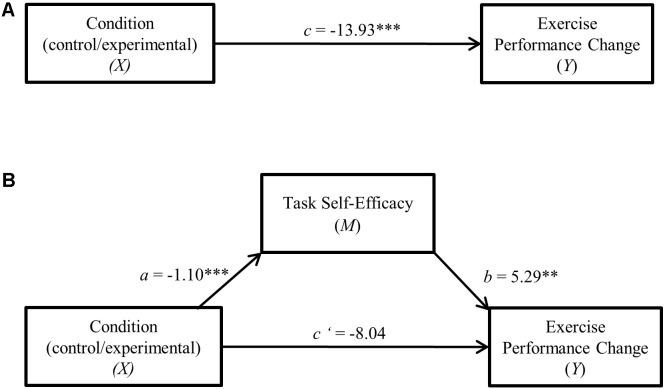
**(A)** Direct effect of cognitive control exertion on residualized change. ^∗∗^*p* < 0.01. **(B)** Cognitive control exertion—residualized change single mediation model. ^∗∗^*p* < 0.01, ^∗∗∗^*p* < 0.001.

Tests for indirect (mediation) effects were assessed using Model 4 in the *PROCESS* software macro (Hayes, 2017). As recommended by Hayes and Scharkow (2013), bias-corrected bootstrap procedures utilizing 10,000 simulations were computed. To evaluate our hypothesis that self-efficacy mediates the effect of cognitive control exertion on exercise performance (raw time to failure change and residualized change), indirect effects analyses were computed. A confidence interval that does not cross 0 indicates a significant (*p* < 0.05) indirect (mediation) effect. Effect size for mediation is reported as the completely standardized indirect effect (*csie*) as it can “generally be used in other situations where it is important to compare indirect effects” (see Preacher and Kelley, 2011, pp. 99–100). Direct effect path coefficients from the mediation models are presented in **Figures 2, 3**.

The secondary hypothesis, investigating whether the change in endurance exercise performance (raw time to failure change and residualized change) following cognitive control exertion is influenced by motor coordination, was evaluated using 2 (LCC/HCC) × 2 (LMC/HMC) univariate factorial ANOVAs. A median split was performed on motor coordination scores (i.e., sex-specific standardized scores) to produce a dichotomized variable representing LMC and HMC. The median value was 48 which resulted in eight participants being removed to produce variables representing LMC and HMC at each level of cognitive control. Effect sizes for the 2 × 2 ANOVAs are reported as partial eta squared $\left(\eta_{p}^{2}\right)$ and the values for small, medium, and large are, respectively, 0.01, 0.06, and 0.14.

## Results

### Potential Covariates

Descriptive statistics, ANOVA summaries, and effect sizes for age, motor coordination, and other potential covariates are shown, by group, in **Table 1**. Analyses revealed no significant differences (*p* > 0.05) between conditions. As previously mentioned, there has been considerable debate regarding the processes that are attributed to negative changes in performance following HCC exertion (see Baumeister and Vohs, 2016b for a review). However, our analyses of potential covariates show that the cognitive control manipulation did not lead to group differences in several proposed variables (i.e., motivation and affect) from various theoretical accounts. Thus, no covariates were included in the main analyses.

**Table 1:** Comparison of age, motor coordination, potential covariates, and manipulation checks by condition.

	Low cognitive control *n* = 33 (13 females) *M* (SD)	High cognitive control *n* = 37 (15 females) *M* (SD)	*F*	*p*	*d*
Age	9.97 (1.91)	10.30 (1.90)	0.52	0.48	0.17
BOT-2 standard score	48.16 (8.49)	48.49 (7.28)	0.03	0.86	0.09
**Potential Covariates**					
Standing long jump (cm)	134.62 (21.36)	139.29 (30.06)	0.55	0.46	0.17
Grip strength (Newtons)	161.06 (51.13)	167.60 (74.76)	0.18	0.68	0.10
50 foot sprint test (seconds)	7.85 (0.81)	7.91 (1.03)	0.09	0.77	0.06
Body Mass Index	17.98 (3.02)	18.26 (3.81)	0.12	0.74	0.08
BRIEF2—BRI	48.40 (7.05)	49.16 (7.13)	0.17	0.68	0.11
BRIEF2—ERI	49.23 (8.26)	51.29 (10.19)	0.75	0.39	0.22
BRIEF2—CRI	52.60 (9.24)	48.87 (9.23)	2.48	0.12	0.40
BRIEF2—GEC	51.07 (8.60)	49.19 (7.27)	0.85	0.36	0.24
IMI effort/importance Trial 1	5.76 (0.98)	5.81 (1.12)	0.04	0.84	0.06
IMI effort/importance Trial 2	6.09 (0.94)	5.92 (1.08)	0.54	0.47	0.17
TEQ Interest/Enjoyment	5.25 (1.10)	5.19 (1.53)	0.04	0.85	0.04
TEQ Perceived Competence	6.00 (1.36)	6.16 (1.36)	0.21	0.65	0.12
TEQ Perceived Choice	5.02 (1.32)	5.07 (1.33)	0.03	0.86	0.04
TEQ Pressure/Tension	3.54 (1.10)	3.50 (1.02)	0.02	0.88	0.04
FS Trial 1-Stroop Δ	-0.18 (1.56)	-0.32 (1.18)	0.30	0.61	0.10
FS Stroop-Trial 2 Δ	0.15 (0.91)	-0.04 (1.26)	0.53	0.47	0.17
Fatigue Trial 1-Stroop Δ	2.27 (16.56)	-0.97 (14.96)	0.74	0.39	0.21
Fatigue Stroop-Trial 2 Δ	-2.10 (12.92)	-2.51 (11.64)	0.02	0.89	0.03
**Manipulation Checks**					
Stroop 1-min trials completed	96.12 (20.52)	91.78 (11.47)	1.25	0.27	0.26
Stroop 1-min RPME	4.70 (2.40)	4.48 (2.71)	0.13	0.72	0.09
Stroop 5-min trials completed	462.91 (96.70)	183.57 (49.94)	237.85	<0.001	3.64
Stroop 5-min errors made	1.09 (1.47)	13.57 (6.07)	132.28	<0.001	2.84
Stroop 5-min RPME	5.49 (2.93)	9.30 (2.49)	34.62	<0.001	1.40


*M, mean; SD, standard deviation; *d*, Cohen’s *d* (effect size); BOT-2, Bruininks-Oseretsky Test of Motor Proficiency (2nd ed.); BRIEF2, Behavior Rating Inventory of Executive Function (2nd ed.); BRI, Behavior Regulation Index; ERI, Emotion Regulation Index; CRI, Cognitive Regulation Index; GEC, Global Executive Composite; IMI, Intrinsic Motivation Inventory; TEQ, Task Evaluation Questionnaire; FS, Feeling Scale; Δ, post-task to post-task change; RPME, ratings of perceived *mental* exertion.*

### Manipulation Checks

Descriptive statistics summarizing Stroop task RPME and performance scores are shown, by group, in **Table 1**. Consistent with the intent of the experimental manipulation, participants in the HCC condition reported greater ratings of mental exertion (*p* < 0.001, *d* = 1.40), completed fewer trials (*p* < 0.001, *d* = 3.64), and made more errors (*p* < 0.001, *d* = 2.84) on the incongruent version of the Stroop task compared to the LCC group.

### Ratings of Perceived Exertion

Descriptive statistics summarizing RPE and RPME scores for exercise performance on Trial 1 and Trial 2 are shown, by group, in **Table 2**. RPE and RPME scores not differ between the groups for either Trial 1 or Trial 2 (*p*-values >0.05), indicating participants in both groups reached equivalent levels of mental and physical exertion on both Trials.

**Table 2:** Handgrip endurance, trial-to-trial change scores, and self-efficacy scores by condition.

	Low cognitive control *n* = 33 *M* (SD)	High cognitive control *n* = 37 *M* (SD)	*F*	*p*	*d*
Trial 1 handgrip score (seconds)	67.40 (26.20)	85.16 (28.32)	7.37	0.008	0.65
Trial 1 RPE	7.65 (2.45)	7.87 (2.73)	0.12	0.73	0.08
Trial 1 RPME	6.36 (3.09)	6.00 (3.07)	0.24	0.62	0.12
Trial 2 handgrip score (seconds)	75.79 (30.75)	75.38 (26.36)	0.01	0.95	0.01
Trial 2 RPE	7.88 (2.60)	8.14 (2.77)	0.16	0.69	0.10
Trial 2 RPME	6.22 (3.22)	5.75 (3.21)	0.36	0.55	0.15
Trial 1 to Trial 2 Δ (seconds)	8.40 (20.30)	-9.78 (13.86)	19.46	<0.001	1.05
Trial 1 to Trial 2 residualized Δ	7.36 (20.40)	-6.56 (12.90)	11.92	0.001	0.82
Task self-efficacy	4.60 (1.19)	3.50 (1.19)	15.04	<0.001	0.92


*M, mean; SD, standard deviation; *d*, Cohen’s *d* (effect size); Δ, trial-to-trial change; RPE, ratings of perceived *physical* exertion; RPME, ratings of perceived *mental* exertion.*

### Primary Analyses

Descriptive statistics summarizing the Trial 1 to Trial 2 raw and residualized endurance handgrip scores are presented in **Table 2**. As seen in the table, there was a 9.78 s reduction in time to failure in the HCC group compared to an 8.40 s increase in the control (LCC) group. A one-way ANOVA revealed significant differences between conditions for both raw (*p* < 0.001, *d* = 1.05) and residualized change scores (*p* < 0.001, *d* = 0.82). Although Trial 1 times were significantly different and not statistically controlled for in the raw change analyses, the residualized change scores are adjusted scores that control for Trial 1 performance in a manner equivalent to ANCOVA (as mentioned above in the Section “Data Analysis”) regardless of condition. However, it is important to note that, residualized change scores are arbitrary values and cannot be interpreted as absolute change in performance (i.e., seconds).

Descriptive statistics summarizing the task self-efficacy scores are also presented in **Table 2**. As predicted, the HCC group reported lower self-efficacy for the second trial compared to controls (*p* < 0.001, *d* = 0.92). To evaluate the hypothesis that task self-efficacy mediates the effect of cognitive control exertion on exercise performance, indirect effects analyses were computed separately for raw time to failure change and residualized change scores. Preliminary examination of the correlation coefficients (Pearson’s *r*) between experimental conditions, time to failure change, and task self-efficacy showed significant (*p* < 0.01) bivariate relationships between all of the variables (see **Table 3**).

**Table 3:** Bivariate correlations (Pearson’s *r*) between experimental condition, handgrip endurance trial-to-trial change score, and task self-efficacy.

	1	2	3
1. Condition (0 = LCC, 1 = HCC)			
2. Trial 1–Trial 2 Δ (seconds)	-0.47^∗∗^		
3. Trial 1–Trial 2 residualized Δ	-0.39^∗∗^	0.94^∗∗^	
4. Task self-efficacy	-0.43^∗∗^	0.48^∗∗^	0.48^∗∗^


*LCC, low cognitive control; HCC, high cognitive control; ^∗∗^*p* < 0.01, Δ, trial-to-trial change.*

In both mediation analyses (depicted in **Figures 2, 3**), performance change score served as the dependent variable with experimental condition (LCC/HCC) specified as the independent variable and task self-efficacy as the mediator. Results indicated a significant indirect (mediation) effect of task self-efficacy for both raw time to failure change (95% C.I. = -11.37, -1.88, *csie* = -0.15) as well as residualized change scores (95% C.I. = -11.41, -1.92, *csie* = -0.16).

### Secondary Analyses

A 2 (LCC/HCC) × 2 (LMC/HMC) univariate ANOVA for raw time to failure change scores showed an overall main effect for condition (*p* < 0.001, $\eta_{p}^{2}$ = 0.27), while the main effect for motor coordination (*p* = 0.11, $\eta_{p}^{2}$ = 0.04) and the condition by motor coordination interaction (*p* = 0.23, $\eta_{p}^{2}$ = 0.02) were not significant.

**FIGURE 4:**
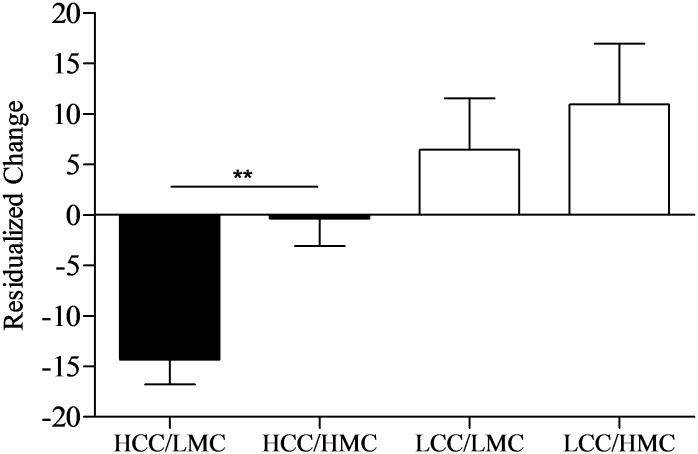
Residualized change scores by levels of cognitive control and motor coordination. HCC, high cognitive control; LCC, low cognitive control; LMC, low motor coordination; HMC, high motor coordination. Error bars represent SE of the mean. ^∗∗^*p* < 0.01.

The 2 × 2 univariate ANOVA for residualized change scores showed significant main effects for condition (*p* < 0.001, $\eta_{p}^{2}$ = 0.20) and motor coordination (*p* < 0.03, $\eta_{p}^{2}$ = 0.08), while the interaction was not significant (*p* = 0.26, $\eta_{p}^{2}$ = 0.02). However, because we hypothesized levels of motor coordination would only affect exercise performance following HCC, independent *t*-tests were computed to evaluate the main effect for motor coordination at each level of cognitive control (depicted in **Figure 4**). As predicted, following HCC, levels of motor coordination had a large effect (*p* = 0.001, *d* = 1.30) on residualized change scores as LMC (*n* = 14) participants experienced greater reductions in residualized change scores when compared to HMC participants (*n* = 20). Alternatively, following LCC, levels of motor coordination had a small effect (*p* = 0.57, *d* = 0.21) on residualized change between LMC (*n* = 15) and HMC (*n* = 14) participants.

## Discussion

The present study investigated the effects of cognitive control exertion and motor coordination on task self-efficacy and exercise performance in children. It was hypothesized that HCC exertion would have negative aftereffects on task self-efficacy and exercise performance, and that task self-efficacy would mediate the effect of cognitive control exertion on exercise performance. We also predicted that children scoring lower on motor coordination would experience greater declines in exercise performance following HCC exertion compared to those with higher motor coordination.

Consistent with our hypotheses, task self-efficacy and exercise performance were negatively affected following the exertion of HCC. Task self-efficacy also mediated the effect of HCC on exercise performance. These findings support previous research showing that the negative aftereffects of HCC, typically seen in young adults (Hagger et al., 2010), also occur in children. However, this is the first study to show the negative aftereffects on task self-efficacy and exercise performance in children. These findings also support emerging research highlighting the influential role of task self-efficacy in explaining performance decrements following the exertion of cognitive control (Chow et al., 2015; Graham and Bray, 2015; Brown and Bray, 2017b; Graham et al., 2017).

As previously mentioned in the introduction, there is a global physical inactivity epidemic occurring among all age groups (Sallis et al., 2016). Although several psychological theoretical models (e.g., Theory of Planned Behavior; Ajzen, 1991) have been used to guide research, a common finding is that despite individuals’ good intentions to be physically active they often do not translate these intentions into behavior, referred to as the “intention-behavior” gap (Rhodes and Bruijn, 2013; also see Sheeran and Webb, 2016). Research based on the Strength Model has proposed, and provided initial evidence, that one of the reasons people fail to translate their intentions into behavior is a result of prior exertions of HCC (Martin Ginis and Bray, 2010; Englert and Rummel, 2016; Pfeffer and Strobach, 2017; Schöndube et al., 2017). Thus, given the global physical inactivity epidemic, alongside self-efficacy’s role in predicting physical activity behavior (McAuley and Mihalko, 1998; Sallis et al., 2016), findings from the present study provide a new avenue of research for understanding inactivity patterns that may also be attributed to reductions in self-efficacy that occur following the exertion of cognitive control on previous, unrelated, tasks.

A recent meta-analysis (Friese et al., 2017) has shown that cognitive control training increases performance on tasks requiring cognitive control. In addition, cognitive control training techniques used in adults to enhance endurance exercise performance (Bray et al., 2015) warrant investigation in children with and without deficits in motor coordination. Further, Bray et al. (2015) suggested that the training regimen used in their study (which required minimal coordination) may have also increased participants’ self-efficacy to exert cognitive control over exercise behavior in general. Studies evaluating the effects of cognitive control training techniques or programs should also investigate the effects of training on self-efficacy.

Although the effect of motor coordination on the cognitive control—performance relationship was a secondary research question, our findings suggest that some children may be more affected by cognitive control exertion than others. Specifically, children scoring lower on motor coordination experienced greater declines in exercise performance following HCC exertion compared to those with higher motor coordination. However, levels of motor coordination were not related to changes in exercise performance following LCC. These results support previous research showing interrelationships between brain areas responsible for cognition and motor behavior (Diamond, 2000; Leisman et al., 2016; Munro et al., 2017). Moreover, they are the first to suggest that neural structures or mechanisms shared between these regions that are temporarily perturbed by cognitive control exertion cause exacerbated performance on simple motor tasks among children low in motor coordination. As such, motor coordination provides a new explanatory mechanism for reductions in physical performance that are characteristically observed following cognitive control exertion (Englert, 2016) and, thus, is worthy of future exploration across different tasks (e.g., cognitively demanding tasks followed by other cognitively demanding tasks) and in different age groups.

**FIGURE 5:**
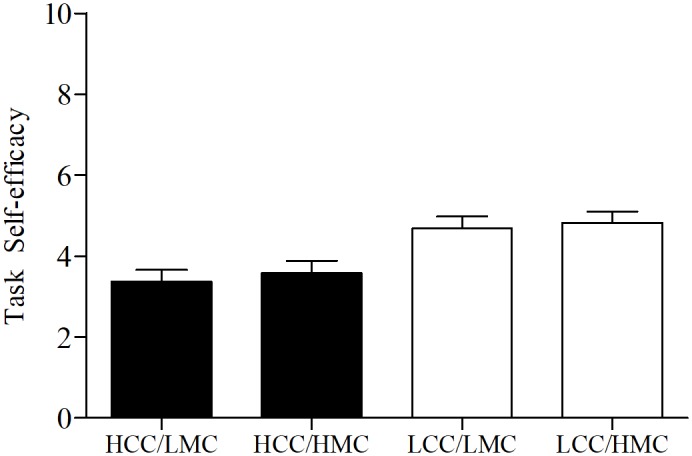
Self-efficacy scores by levels of cognitive control and motor coordination. HCC, high cognitive control; LCC, low cognitive control; LMC, low motor coordination; HMC, high motor coordination. Error bars represent SE of the mean.

Our secondary (exploratory) analyses also provide insights into the unique relationships between motor coordination, task self-efficacy, and exercise performance following cognitive control exertion. Specifically, we found that self-efficacy mediated the cognitive control—performance relationship. We also found children scoring lower on motor coordination experienced greater declines in exercise performance following HCC exertion compared to those with higher motor coordination (seen in **Figure 4**). However, self-efficacy scores across levels of motor coordination were nearly equal at each level of cognitive control (depicted in **Figure 5**). One interpretation of these findings is that participants higher in motor coordination may have drawn on their greater self-efficacy beliefs to persist longer at the task as it became more difficult to perform. Indeed, based on the change in exercise performance across levels of cognitive control and motor coordination (**Figure 4**) alongside self-efficacy scores (**Figure 5**), our findings suggest that self-efficacy beliefs among those high in motor coordination may buffer against the negative aftereffects of HCC.

Although the above interpretation is plausible, it should be noted that the administration of the self-efficacy measure occurred immediately prior to the second endurance trial and does not provide data on in-task efficacy beliefs. As the handgrip task was a novel task for all participants, and the fact that they were not provided with feedback about their performance on the first endurance trial, it is also possible participants with lower levels of motor coordination might have overestimated their ability. Indeed, people often have a hard time gauging their self-efficacy for performance when a task is novel without any feedback regarding their previous performance (Bandura, 1997). Research also suggests that children have a hard time gauging their efficacy beliefs for physical tasks across a variety of situations (Horn and Weiss, 1991). Yet, we found that participants uniformly reported lower levels of self-efficacy following HCC when compared to LCC, regardless of levels of motor coordination. Thus, following HCC, we believe participants with higher levels of motor coordination relied more on their in-task efficacy beliefs to maintain task performance whereas efficacy beliefs deteriorated as the task progressed among those with lower levels. However, future research is needed utilizing more ecologically valid sport and exercise tasks that children may have had previous experience with (i.e., running, jumping, throwing, catching, etc.) to allow them to more accurately gauge their efficacy beliefs following HCC. Future research is also encouraged to assess in-task self-efficacy beliefs (alongside motor coordination) following the exertion of HCC.

In addition to further exploration of motor coordination effects associated with cognitive control exertion, it is important to think about neurophysiological processes that facilitate the *communication* between regions governing cognition and motor behavior. It is plausible that performing the incongruent version of the Stroop task (between the endurance trials) perturbed the connection between brain regions governing cognition and motor behavior in a way that altered the efficiency of the signal. Dopamine is a neurotransmitter known to facilitate the communication between brain regions governing cognition and motor behavior (for reviews see Nieoullon, 2002; Cools, 2016). Therefore, performing the incongruent Stroop task may have either prevented dopamine levels from stabilizing (i.e., returning to baseline levels), altered the efficiency of dopaminergic neurons enabling cognition and motor behavior, or led to subsequent brain-based allocation priorities (e.g., Beedie and Lane, 2012); any or all of which could contribute to negative changes in exercise performance. Given individuals with cognitive and motor deficits have dysregulated dopaminergic pathways (Diamond, 2000; Robinson and Gradinaru, 2018); findings from the present study suggest dopaminergic processes warrant further attention when investigating the relationships between cognition and motor behavior.

Another possible avenue for future consideration in efforts to understand how cognitive control processes may interact with motor control relates to previous research indicating there is an accumulation of Amyloid-β peptides in neural tissue resulting from the prolonged exertion of cognitive control (Holroyd, 2016). Amyloid-β peptide accumulation is thought to trigger interoceptive sensations of fatigue (Holroyd, 2016) that subsequently influence self-efficacy to exert cognitive control (Graham et al., 2017, p. 84). Yet, the dysregulation of dopamine following HCC has been discussed as being subjectively experienced (Westbrook and Braver, 2016) and, in turn, may also negatively influence self-efficacy to exert cognitive control. As such, a pertinent theoretical question relates to self-efficacy’s influence on neurophysiological processes governing cognitive control. As Graham and Colleagues alluded (2017, p. 84), the neurophysiological changes resulting from the exertion of cognitive control are largely automatic, and thus, self-efficacy’s role in guiding subsequent behavior becomes that more interesting. For instance, the neurophysiological alterations in dopamine and Amyloid-β peptides may serve as a signal that the system is perturbed in a manner indicative of inability. Alternatively, “*self-efficacy may be a motivational intersection where neurophysiological processes, affective sensations, and situational perceptions combine to determine how much self-control one will ultimately invest in a task*” (Graham et al., 2017, p. 84). We agree with the later, self-efficacy’s role in the cognitive control—performance relationship is most likely more complicated than a simple indicator of a perturbed system and worthy of continued investigation.

Given the above, and previous research showing self-efficacy is negatively affected following HCC (Chow et al., 2015; Graham and Bray, 2015; Brown and Bray, 2017b; Graham et al., 2017), it seems plausible that self-efficacy may also act as a mechanism that could alter neurophysiological functioning and, in turn, affect performance on tasks requiring cognitive control. Bandura (1997) suggests that self-efficacy beliefs are influenced by four primary sources: past performance mastery, vicarious experiences, verbal persuasion, and physiological/affective states. However, research also suggests that self-efficacy can influence (i.e., mediate) several neurophysiological processes and, ultimately, subsequent behavior (Bandura, 1997, “*Biological Effects of Perceived Self-Efficacy*,” pp. 262–278; Schönfeld et al., 2017). Thus, the manipulation of efficacy beliefs following the exertion of HCC may help individuals overcome the negative aftereffects on performance through changes in neurophysiological processes that facilitate effective cognitive control.

Recent research by Luethi et al. (2016) has shown that the neurophysiological processes governing cognitive control can be enhanced following HCC. For instance, Luethi et al. (2016) showed that monetary incentives not only counter the negative effects of HCC exertion on task performance but, more importantly, result in increased blood flow (using fMRI) within regions responsible for cognitive control and motor behavior. These findings are particularly intriguing as they show neurophysiological processes are affected differently following the prolonged exertion of cognitive control and, specifically, in a manner suggestive that subjective experiences can alter subsequent neurophysiological functioning. Recent research has also shown that monetary incentives counter the negative aftereffects of HCC on endurance handgrip performance through alterations in neuromuscular activation patterns (Brown and Bray, 2017a). Although self-efficacy was not directly assessed in the studies by Luethi et al. (2016) and Brown and Bray (2017a), previous research has shown that monetary incentives increase self-efficacy and, in turn, task performance (for a review see Bonner and Sprinkle, 2002). Thus, in the studies reviewed above, it is plausible that monetary incentives increased participants’ self-efficacy to exert cognitive control, and importantly, subsequently altered neurophysiological processes enabling effective cognitive control.

There are several novel findings from the present study that provide exciting avenues for research investigating the cognitive control—performance relationship, however, there are also limitations. Although the exercise task and cognitive control manipulation were suitable for a controlled laboratory experiment, future research needs to test these hypotheses using more ecologically valid exercise and cognitive control tasks. It is important to note that our secondary analyses were exploratory and future research is encouraged to replicate our study design and generate sample-size calculations based on our findings.

We also investigated motor coordination abilities in a typically developing sample. However, future research should replicate our study in clinical populations (e.g., developmental coordination disorder (DCD) and attention deficit hyperactivity disorder (ADHD)) where comorbidity between impairments in motor coordination and cognitive control exist. For instance, children with DCD, a disorder that affects fine and gross motor skills, also show deficits in cognitive control (Cairney, 2015). Similarly, children with ADHD, a disorder that affects cognitive control, also show deficits in motor coordination (e.g., Barkley, 1990). In fact, approximately half of children diagnosed with ADHD are also diagnosed with DCD (Martin et al., 2006). As many neurodevelopmental and psychiatric disorders persist into adolescence and adulthood (e.g., Ferdinand and Verhulst, 1995; Costello et al., 2003; Kirby et al., 2008), future research is also encouraged to replicate the present study among different age groups while controlling for the potential effect of various medications (e.g., Methylphenidate) and secondary mental health outcomes (i.e., anxiety and depression) often associated with these disorders (e.g., Cairney et al., 2013). Thus, the effects seen in the present study may be even greater among children with neurodevelopmental and related psychiatric disorders.

We encouraged future research to assess in-task self-efficacy; however, it is also important to investigate whether feedback (both in-task and prior to task completion) may affect self-efficacy for exercise performance following HCC. Emerging research suggests children often rely on feedback from influential others (i.e., coaches, parents, peers) to gauge their self-efficacy when performing various sport and exercise tasks (Jackson et al., 2014; Saville et al., 2014). Thus, it is possible that self-efficacy enhancing feedback (i.e., “*I believe in you*,” “*I know you can do this*”) may increase task self-efficacy and, in turn, counter the negative aftereffects of HCC. Finally, although discussion pertaining to alterations in brain (see Luethi et al., 2016) and muscle (see Brown and Bray, 2017a,b) activation was supported by previous research, future research is needed to investigate both brain and muscle activation patterns concurrently to further understand the unique contributing neurophysiological processes that govern cognitive control over exercise behavior.

Research should also examine specific sub regions within areas governing cognition (i.e., prefrontal cortex) and motor behavior (i.e., motor cortex and cerebellum) using advanced neuroimaging techniques. For instance, research has shown that endurance handgrip squeezing elicits cortical activation within both the rostral prefrontal cortex and primary sensori-motor areas (see Derosière et al., 2014). Importantly, Derosière et al. (2014) also found higher activation within the right rostral prefrontal cortex, an area known to be responsible for sustained attention which contributes to cognitive control. However, research has also shown that performance of the Stroop task elicits cortical activation within sub regions of the prefrontal cortex (i.e., lateral and medial frontal regions), primary motor cortex (i.e., Brodmann area 6), among other regions (anterior cingulate) that contribute to sustained attention, inhibition, and motor modulation (Leung et al., 2000). It is possible that a cognitive control manipulation requiring primarily working memory capacity, rather than inhibition and sustained attention which are primarily utilized during the Stroop task (see Hofmann et al., 2012), may show a different pattern of findings (across levels of motor coordination) based on various neural contributions required for task performance (Pessoa et al., 2002). Although Pessoa et al. (2002) also showed that performing a working memory task elicits premotor activation. Nevertheless, it is important for future research to examine the specific contributions within sub regions governing cognition and motor behavior to provide a greater understanding of the interrelations between cognition, motor behavior, and task performance following the exertion of HCC.

The present study has provided the first evidence of the negative aftereffects of HCC exertion on self-efficacy and exercise performance in children. Interestingly, the negative aftereffects on exercise performance were the greatest among children scoring lower on motor coordination. These results highlight the unique relationship between brain areas governing cognitive control and motor behavior that have been largely studied separately. Thus, motor coordination provides an exciting new mechanism for the negative aftereffects of HCC that should be studied across a range of cognitive and physical behaviors.

## Author Contributions

JG formulated the research question and study design, and was primarily responsible for data collection, analysis, and writing of the manuscript. YCL assisted with data collection and provided valuable comments during manuscript preparation. SB and JC contributed to study design, data analysis, interpretation of results, and writing of the manuscript. All the authors approved the final manuscript as submitted and agreed to be accountable for all aspects of the work.

## Conflict of Interest Statement

The authors declare that the research was conducted in the absence of any commercial or financial relationships that could be construed as a potential conflict of interest.

## Footnotes

1 www.random.org
